# Consumption of ready-made meals and increased risk of obesity: findings from the Observation of Cardiovascular Risk Factors in Luxembourg (ORISCAV-LUX) study

**DOI:** 10.1017/S0007114514003468

**Published:** 2014-12-09

**Authors:** Ala'a Alkerwi, Georgina E. Crichton, James R. Hébert

**Affiliations:** 1 Centre de Recherche Public Santé, Centre d'Etudes en Santé, Grand-Duchy of Luxembourg, 1A rue Thomas Edison, L-1445Strassen, Luxembourg; 2 Nutritional Physiology Research Centre, University of South Australia, Adelaide, SA, Australia; 3 Cancer Prevention and Control Program, University of South Carolina, Columbia, SC29208, USA; 4 Department of Epidemiology and Biostatistics, Arnold School of Public Health, University of South Carolina, Columbia, SC29208, USA

**Keywords:** Ready-made meals, Cardiovascular risk, Obesity

## Abstract

The consumption of ready-made meals, such as pre-packaged dishes, available at grocery stores and fast-food restaurants, is a habit related to our modern fast-paced lives. No study has examined the association of daily ready-made meal consumption with diet quality or health-related outcomes. The present study aimed to investigate the association between self-reported ready-made meal consumption and diet quality, as measured by compliance with dietary recommendations and with a set of adiposity measures, in a nationally representative sample of 1352 subjects, aged 18–69 years, participating in the nationwide population-based ORISCAV-LUX (Observation of Cardiovascular Risk Factors in Luxembourg) survey. The daily consumption of ready-made meals was calculated as follows: frequency of consumption × portion size × number of portions consumed. The sum of the daily consumption values of the eleven pre-packaged dishes included in the FFQ represented the total daily consumption of ready-made meals (g/d) for each participant. About 97 % of the participants reported daily consumption of ready-made meals. The intake was highly prevalent in men living alone and varied according to education level. Ready-made meal consumption provided >7 % of total daily energy. The fractions (%) of macro- and micronutrients derived from daily consumption of ready-made meals varied from 10 % for total cholesterol to 0·65 % for total fibre. Increased consumption of ready-made meals was found to be independently associated with abdominal obesity. On controlling for age, sex, socio-economic status and lifestyle factors, daily consumption of ready-made meals was found to be associated with higher energy intake and with poor compliance with national nutritional recommendations, and hence it could plausibly increase the risk of central obesity and fat deposition.

Obesity has become a worldwide public health epidemic, associated with numerous health problems including type 2 diabetes, heart disease and stroke^(^ ^1^ ^)^. The substantial costs associated with the prevention and treatment of these conditions are taking a heavy toll on healthcare systems around the world, including Luxembourg, where 21 % of the population is obese^(^ ^2^ ^)^. Current research reveals that several environmental factors could be fuelling the obesity epidemic; diet quality is coming to the fore as a major modifiable factor.

In our modern fast-paced lives, less time is dedicated to the preparation of meals. Consequently, the consumption of ready-made meals, such as ready-to-heat pre-packaged dishes available at grocery stores and fast-food restaurant items, has increased^(^ ^3^ ^)^. These foods represent a quick and easy alternative to home-prepared meals, as they are sold in a partly or completely cooked form and are ready to eat within minutes. Before reaching the consumer, ready-made meals already undergo some form of processing to ensure food safety or hygiene or to enhance palatability, texture or flavour. Processing may involve the addition of other foods or ingredients, such as preservatives, as well as heating, cooling or pressure-cooking. The distinction from raw or unprocessed foods or meals prepared at home is that the consumer cannot control the nutritional quality of the basic ingredients or the amount of added sugars or fats used in these dishes.

This shift in dietary habits towards increasing consumption of ready-made meals is concomitant with the rise in the prevalence of obesity^(^ ^1^ ^,^ ^3^ ^)^. The consumption of fast foods or meals outside the home has consistently been linked to the consumption of more energy and saturated fat and fewer fruits and vegetables^(^ ^4^ ^–^ ^6^ ^)^. For several years, researchers have focused on rising fast-food consumption as a key contributor of weight gain among adults^(^ ^7^ ^–^ ^9^ ^)^ and childhood obesity^(^ ^10^ ^)^. A positive association between eating out of home and weight gain has been confirmed by two systematic reviews^(^ ^5^ ^,^ ^9^ ^)^.

Moreover, high consumption of pre-prepared or pre-packaged dishes (hereafter referred to as ready-made meals) could be problematic. These ready-made meals can be consumed at fast-food restaurants or purchased and then eaten at home. Poti & Popkin^(^ ^11^ ^)^ reported that food sources including both fast foods and store-prepared foods significantly affect the daily energy intake of American children and hence their body weight. These findings are consistent with our hypothesis that the emergent patterns of eating ready-made meals, whether at home or at fast-food eateries, have an impact on body weight control and, consequently, present a new challenge threatening the nutritional quality of the consumer's diet. So far, it is unknown whether high consumption of ready-made meals, as measured in g/d, is independently associated with weight status among adults or the quality of their diet. The aim of the present study was to investigate the association between self-reported ready-made meal consumption and diet quality, as measured by compliance with dietary recommendations and with a set of adiposity measures, in a nationally representative sample of adult residents of Luxembourg.

## Methods

### Study population

The Observation of Cardiovascular Risk Factors in Luxembourg (ORISCAV-LUX) is a cross-sectional, population-based survey of cardiovascular risk factors in the adult population of Luxembourg^(^ ^2^ ^)^. A systematic random sample of 1432 subjects selected from the national health insurance registry, according to age, sex and district, participated in the survey. The distribution of selected subjects in each stratum was proportional to their distribution in the source population. The participants visited the nearest study centre after telephone appointment. During the interview, the trained research staff provided the participants with information on the study objectives, gave detailed instructions on how to complete the FFQ, helped them complete questionnaires related to dietary information, and then checked the correctness of the answered questionnaires. Further information regarding demographic, socio-economic and lifestyle factors was collected as well as direct blood pressure and anthropometric measurements were taken. More details of the ORISCAV-LUX study design, data collection methods, sample selection and representativeness have been reported previously^(^ ^2^ ^,^ ^12^ ^)^. For the present study, a set of 1352 subjects was available after eliminating those with missing data on dietary habits or adiposity.

### Assessment of dietary intake

Dietary intake was assessed over the previous 3 months, using a semi-quantitative FFQ, which was validated against nutritional biomarkers^(^ ^13^ ^)^, and a 3 d dietary record^(^ ^14^ ^)^. The FFQ comprised 134 items, categorised into nine major food groups: grains; fruits; vegetables; meat–poultry–fish–eggs; dairy products; fats; beverages (alcoholic and non-alcoholic); ready-made meals; miscellaneous items (online supplementary Table S1). The participants reported the frequency of consumption of each food or beverage from six levels, ranging from ‘rarely or never’ (i.e. less than once per month) to ‘two or more times per day’. Nutrient intakes were calculated by multiplying the consumption frequency of each food by the nutrient content of the specified portions. Nutrient intake values were obtained from the French SUpplémentation en VItamines et Minéraux Anti-oXydants (SU.VI.MAX) Food Composition Database^(^ ^15^ ^)^.

### Assessment of ready-made meal consumption

The FFQ was designed to include ‘ready-made meals’ as an individual food group. The term ‘ready-made meals’ refers to dishes prepared away from home and pre-packaged in a disposable serving tray, which needs only heating before serving. The research nurse clearly explained to the participants that this food group indicates the mixed pre-prepared dishes, processed by food manufacturers or by caterers, such as burger outlets. These foods were ready-to-heat meals typically purchased from food-service establishments, grocery stores or supermarkets (in the form of frozen, partially or completely cooked pre-packaged meals). A total of eleven individual dishes were included within this food category, including typical ‘fast foods’ as well as a number of prepared dishes representing different cuisine specialties. The eleven included foods were hamburgers, pizza, paella, stuffed pasta (ravioli, lasagne), quiche/savoury tart, prepared dishes with cod (bacalhõ/Portuguese specialty), cabbage with sausages (sauerkraut/German specialty), smoked pork with beans (local Luxembourg specialty), spring rolls (Indonesian specialty), cheese crepes and *Reisling pâté* (kind of pasta/local Luxembourg specialty), spring rolls (Indonesian specialty) and cheese crepes.

The daily consumption value for every item (prepared dish) was calculated as follows: frequency of consumption (times/d) × portion size (g) × number of portions consumed. The sum of the daily consumption values of the eleven items represented the total daily consumption of ready-made meals (g/d) for each participant.

### Measurement of anthropometric and obesity parameters

All clinical procedures were performed in accordance with the ORISCAV-LUX standardised operating protocol. Body weight was measured using a digital column scale (Seca^®^ 701; Seca), recorded to the nearest 0·1 kg, with the participants in barefoot and wearing light clothing. Standing body height was recorded to the nearest 0·2 cm with a portable wall stadiometer (Seca). Waist circumference (WC, cm) was measured at the level midway between the twelfth rib and the uppermost lateral border of the iliac crest during normal expiration. WC was measured to the nearest 0·2 cm in standing position, using a flexible, non-distensible tape without exertion of pressure on the tissues.

BMI was calculated as weight in kg divided by height in m^2^. Global obesity was defined as BMI ≥ 30 kg/m^2(^ ^16^ ^)^, while abdominal obesity was defined as WC ≥ 102 cm for men and WC ≥ 88 cm for women^(^ ^17^ ^)^. Body surface area (BSA) was calculated according to the Mosteller formula^(^ ^18^ ^)^:
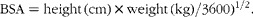



The BSA was considered ‘normal’ if the mean value was ≤ 1·91 m^2^ for men and ≤ 1·71 m^2^ for women^(^ ^19^ ^)^.

### Assessment of covariates

Information on socio-economic (education level, income and marital status) and lifestyle (smoking status and physical activity) factors was obtained using a self-administered questionnaire. Education level, based on the highest diploma obtained, was classified into three categories: highest ‘tertiary level’, equivalent to university or higher qualification; ‘secondary level’, equivalent to classical or technical qualification; ‘primary level’, corresponding to non-academic qualification, but at least first 9 years of mandatory schooling. Income was classified into ‘living above’ or ‘below’ the poverty threshold. Marital status was categorised as ‘live alone’ or ‘live with partner’. Physical activity during the last 7 d before the interview was assessed using the International Physical Activity Questionnaire, which categorised the population into three groups: ‘physically inactive’; ‘moderately active’; ‘active’^(^ ^20^ ^)^. The participants were classified as ‘smokers’ or ‘non-smokers’ based on their smoking status. Further details regarding the collection of information on these variables have been published elsewhere^(^ ^21^ ^)^.

The total daily intakes of the eight other food groups in the FFQ, i.e. grains (g/d), fruits (g/d), vegetables (g/d), added fats (g/d), meat–poultry–fish–eggs (g/d), dairy products (servings/d), alcoholic and non-alcoholic beverages (g/d), and miscellaneous items (g/d), as well as total energy intake (Kcal/d), were calculated (online supplementary Table S1).

### Ethical aspects

The present study was conducted according to the guidelines laid down in the Declaration of Helsinki, and all procedures involving human subjects were approved by the National Research Ethics Committee and the National Commission for Private Data Protection. Written informed consent was obtained from all the participants.

### Statistical analyses

The characteristics of participants by daily consumption of ready-made meals (in two categories: above and below the median) were compared using the χ^2^ test. ANOVA was used to assess differences in the mean and standard deviation for a set of dietary continuous variables according to daily consumption of ready-made meals. The χ^2^ and binary logistic regression analyses were carried out to examine the association between consumption of ready-made meals and meeting the national dietary recommendations.

Several multivariable logistic regression models were fit to investigate the association of ready-made meal consumption with obesity, as measured by three indicators (BMI, WC and BSA). Model 1 was adjusted for age, sex and socio-economic factors (education level, income and marital status). Model 2 was further adjusted to include lifestyle factors (physical activity and smoking status). Model 3 also included additional dietary factors that could confound associations or serve as mechanisms linking ready-made meal consumption to weight gain, including daily intakes of grains, fruits and vegetables, meat–poultry–fish–eggs, dairy products, added fats, alcoholic and non-alcoholic beverages, and miscellaneous items and total energy intake. This series of models allows for exploring the individual and combined impacts of these potential mediators (socio-economic status, physical activity and dietary factors) on the association between obesity and ready-made meal consumption.

A fourth model was fit for BSA, with additional controlling for height. The robustness of the results of the present study was established by performing two sensitivity analyses: one conducted after excluding the outliers (eight cases, constituting 0·6 % of the sample who were consuming >400 g/d of ready-made meals) and the other conducted to test sex and age interactions with ready-made meal consumption.

Results were considered significant at a two-tailed type 1 (i.e. α) error rate of 5 % (*P*< 0·05). All statistical analyses were performed using PASW^®^ for Windows^®^ version 18.0 software (formerly SPSS Statistics, Inc.)

## Results

### Characteristics of the participants according to daily consumption of ready-made meals

As the intake was skewed, the median value (70 g/d) was more accurate and meaningful than the mean (92 g/d). Low consumers had intakes < 70 g/d. High consumers had intakes ≥ 70 g/d, up to a maximum of 572 g/d.

Compared with low consumers, high consumers were more likely to be males (60 %) and living alone (32·5 %). The daily consumption of ready-made meals varied considerably according to education level (*P*= 0·024), but not according to smoking status or physical activity (Table 1).

**Table 1:** Characteristics of the study participants according to daily consumption of ready-made meals, ORISCAV-LUX (Observation of Cardiovascular Risk Factors in Luxembourg) survey, 2007–8 (Number of participants and percentages; mean values with their standard errors, *n* 1352)

	Daily consumption of ready-made meals
	Below the median ( < 70 g/d) (low consumers)	Above the median ( ≥ 70 g/d) (high consumers)	
	*n*	%	*n*	%	*P*
Range of intake (g/d)	0·00–70·0	70·1–571·9	
Ready-made meal consumption	679	50·2	673	49·8	
Age (years)					< 0·0001
Mean	47·04	41·53	
se	0·49	0·50	
Demographic and socio-economic characteristics
Sex					< 0·0001
Male	256	37·7	401	59·6	
Female	423	62·3	272	40·4	
Education level					0·024
Primary	195	39·6	156	31·8	
Secondary	173	35·1	180	36·7	
Tertiary	125	25·4	145	31·4	
Live alone	175	25·8	219	32·5	0·004
Income					0·08
Above the poverty threshold	452	76·7	470	80·3	
Lifestyle factors					
Smokers	134	19·7	154	22·9	0·09
Physical activity					0·23
Inactive	123	19·1	100	15·5	
Moderately active	174	27·0	185	28·6	
Active	347	53·9	361	55·9	
Global obesity	156	23·0	148	22·0	0·35
Abdominal obesity	235	34·7	197	29·3	0·019
Body surface area	416	61·4	457	67·9	0·007

### Ready-made meal consumption and other dietary variables

Compared with low consumers, high consumers had significantly higher mean intakes of total energy, grains, meat–poultry–fish–eggs, alcoholic drinks and miscellaneous items. Lower intakes of fruits and vegetables were also observed among high consumers, although this association was not statistically significant. For each 1 g/d of ready-made meal consumption, there was an increase of 23·0 kJ/d (5·5 kcal/d) in energy intake, after controlling for age, sex, socio-economic status and lifestyle factors (Table 2).

**Table 2:** Description and multivariable regression estimates of dietary variables with regard to daily consumption of ready-made meals, ORISCAV-LUX (Observation of Cardiovascular Risk Factors in Luxembourg) survey, 2007–8 (Mean values with their standard errors)

	Daily consumption of ready-made meals
	Below the median (low consumers)	Above the median (high consumers)	Total		Multivariable regression estimates*	
Dietary variables	Mean	se	Mean	se	Mean	se	*P*	β	se	*P*
Ready-meal consumption (g/d)	38·3	0·8	145·6	3·0	91·7	2·1	< 0·0001			
Total energy intake							< 0·0001	5·50	0·41	< 0·0001
kcal/d	2083·2	30·0	2758·7	37·0	2419·4	25·5				
kJ/d	8716·1	125·5	11 542·4	154·8	10 122·8	106·7				
Fruit and vegetable intake (g/d)	577·0	16·7	540	13·7	558·6	10·8	0·088	0·28	0·19	0·14
Grain intake (g/d)	285·5	7·3	331·2	6·4	308·2	4·9	< 0·0001	0·28	0·08	0·001
Meat–poultry–fish–egg intake (g/d)	163·4	3·6	229·6	4·8	196·3	3·1	< 0·0001	0·38	0·05	< 0·0001
Dairy product intake (servings/d)	2·0	0·1	2·2	0·1	2·10	0·1	0·08	0·003	0·001	0·001
Added fat intake (g/d)†	46·8	1·4	56·9	1·6	51·8	1·1	< 0·0001	0·10	0·01	< 0·0001
Non-alcoholic drink intake (g/d)	1845·4	32·6	1871·5	32·2	1858·4	22·9	0·57	0·47	0·42	0·26
Alcoholic drink intake (g/d)	126·2	8·2	170·8	11·1	148·4	6·9	0·001	0·13	0·13	0·33
Miscellaneous item intake (g/d)	56·5	2·5	77·43	2·7	66·9	1·9	< 0·0001	0·18	0·04	< 0·0001

* Models were adjusted for age, sex (male or female), education level (primary, secondary or tertiary), marital status (live alone or live with partner), income (above or below the poverty threshold), physical activity (active, moderately active or inactive) and smoking status (smoker or non-smoker). Daily ready-made meal consumption (explanatory variable) was used as a continuous variable. The estimates are specifically for a 1 g/d increase in ready-made meal consumption.

† Refers to the intake of added fats and oils.

Across the entire sample, >7 % of total daily energy intake was derived from ready-made meals. The fractions (%) of macronutrients derived from daily consumption of ready-made meals ranged from 0·65 % for total fibre to 10 % for total cholesterol (Table 3).

**Table 3:** Fractions of macronutrients derived from daily consumption of ready-made meals*

	Daily consumption of ready-made meals
Dietary variables	Below the median (low consumers)	Above the median (high consumers)	Total	*P*
Total energy (%)	3·7	0·09	10·5	0·20	7·1	0·14	< 0·0001
Total fat (%)	4·9	0·13	13·0	0·25	8·9	0·17	< 0·0001
SFA (%)	5·4	0·14	14·0	0·27	9·7	0·19	< 0·0001
Fibre (%)	0·3	0·01	1·0	0·02	0·7	0·02	< 0·0001
Total protein (%)	4·6	0·11	12·3	0·23	8·4	0·16	< 0·0001
Vegetal protein	3·0	0·09	9·7	0·23	6·3	0·15	< 0·0001
Animal protein	4·9	0·13	12·3	0·27	8·6	0·18	< 0·0001
Total cholesterol (%)	6·1	0·15	14·1	0·27	10·1	0·19	< 0·0001
Total carbohydrates (%)	2·9	0·08	8·7	0·19	5·8	0·13	< 0·0001

* Calculated as the intake of each nutrient derived from ready-made meals/the total intake of the same nutrient × 100.

### Ready-made meal consumption and dietary recommendations

Generally, the compliance with national nutrient-based recommendations was remarkably lower in participants who consumed >70 g/d of ready-made meals (high consumers) than in those with lower intakes. Higher consumers of ready-made meals had significantly less chance of being compliers of recommendations, with regard to most of the nutrients (including carbohydrates, total fat and SFA). With respect to food-based recommendations, high consumers of ready-made meals were significantly less likely to achieve the fruit and vegetable intake goals compared with the low consumers (55·4 *v.* 60·8 %, respectively; *P*= 0·03). A similar finding was recorded for the goal of eating meat–poultry–fish–eggs 1–2 times/d (46·8 *v.* 55·2 %; *P*= 0·001). These findings remained significant after controlling for age, sex, socio-economic status and lifestyle factors, except for fruit and vegetable intake, where the association became non-significant (Table 4).

**Table 4:** Association between meeting nutrient-based and food-based recommendations and daily consumption of ready-made meals, ORISCAV-LUX (Observation of Cardiovascular Risk Factors in Luxembourg) survey, 2007–8 (Number of participants and percentages; multivariable-adjusted odds ratios and 95 % confidence intervals)

		Daily consumption of ready-made meals
		Below the median (low consumers)	Above the median (high consumers)		Multivariable regression estimates*	
	National intake goals	*n*	%	*n*	%	*P*	OR	95 % CI	*P*
Nutrient-based recommendations									
Carbohydrates	>45 % E	286	42·7	192	28·7	< 0·0001	0·986	0·981, 0·991	< 0·0001
Total fat†	≤ 35 % E	254	38·0	153	22·9	< 0·0001	0·991	0·989, 0·994	< 0·0001
SFA	≤ 10 % E	123	18·4	46	6·9	< 0·0001	0·986	0·981, 0·991	< 0·0001
Total protein	15–20 % E	598	88·9	608	91·6	0·057	1·002	0·998, 1·006	0·38
Food-based recommendations									
Grain products‡	≥ 3 servings/d	247	36·4	246	36·6	0·50	1·001	0·999, 1·003	0·17
Dairy products	≥ 3 products/d	181	26·7	191	28·4	0·26	1·003	1·00, 1·005	0·024
Meat–poultry–fish–eggs	1–2 times/d	375	55·2	315	46·8	0·001	0·998	0·996, 1·000	0·031
Fruits and/or vegetables	≥ 5 servings/d	373	60·8	352	55·4	0·030	1·000	0·998, 1·002	0·90
Non-alcoholic drinks	≥ 1·5 litres/d	444	65·4	435	64·6	0·41	1·001	0·999, 1·003	0·51

%E, percentage of total daily energy intake.

* Models were adjusted for age, sex (male or female), education level (primary, secondary or tertiary), marital status (live alone or live with partner), income (above or below the poverty threshold), physical activity (active, moderately active or inactive) and smoking status (smoker or non-smoker). Daily ready-made meal consumption (explanatory variable) was used as a continuous variable. The estimates are specifically for a 1 g/d increase in ready-made meal consumption.

† Refers to the total fat in the diet (oils and fats added and present in the foods).

‡ Grain products refer to all types of bread, cereals, muesli, pastries, potatoes, rice, pasta and pulses.

### Multivariable modelling of global and abdominal obesity

Controlling for demographic, socio-economic, and lifestyle factors and dietary variables, three successive models were fit to assess the association of ready-made meal consumption with obesity (Table 5). Higher daily consumption of ready-made meals was significantly associated with abdominal obesity, as measured by WC, after full adjustment for all potential confounding factors, including age, sex, and socio-economic, behavioural and dietary factors. However, no significant independent association was detected between ready-made meal consumption and global obesity, as measured by BMI. Although higher BSA was associated with high consumption of ready-made meals, this association became statistically non-significant after further controlling for height.

**Table 5:** Association of abdominal obesity, global obesity and body surface area with daily consumption of ready-made meals based on data from 1352 subjects from the ORISCAV-LUX (Observation of Cardiovascular Risk Factors in Luxembourg) survey (Multivariable-adjusted odds ratios and 95 % confidence intervals)*

	Daily consumption of ready-made meals
	OR	95 % CI	*P*
Abdominal obesity			
Model 1†	1·003	1·000, 1·005	0·021
Model 2‡	1·002	1·000, 1·005	0·038
Model 3§	1·003	1·000, 1·006	0·021
Global obesity			
Model 1	1·001	0·998, 1·003	0·53
Model 2	1·000	0·998, 1·003	0·75
Model 3	1·002	0·999, 1·005	0·29
Body surface area			
Model 1	1·004	1·001, 1·006	0·002
Model 2	1·003	1·001, 1·005	0·007
Model 3	1·003	1·001, 1·005	0·018
Model 4∥	1·002	0·999, 1·005	0·14

* OR and 95 % CI of obesity were derived from binary logistic regression.

† Model 1 was adjusted for age, sex (male or female), education level (primary, secondary or tertiary), marital status (live alone or live with partner), and income (above or below the poverty threshold).

‡ Model 2 was additionally adjusted for physical activity (active, moderately active or inactive) and smoking status (smoker or non-smoker).

§ Model 3 was additionally adjusted for dietary variables, including total energy intake and daily intakes of fruits and vegetables, grains, alcoholic and non-alcoholic beverages, dairy products, added fats and miscellaneous items.

∥ Model 4 (body surface area) was additionally adjusted for height. Daily ready-made meal consumption (explanatory variable) was used as a continuous variable. The estimates are specifically for a 1 g/d increase in ready-made meal consumption.

### Sensitivity analyses

Exclusion of the outliers (eight cases, constituting 0·6 % of the sample who were consuming >400 g/d of ready-made meals) did not alter the findings. There were no sex and age interactions with ready-made meal consumption.

## Discussion

Over recent years, there have been major alterations in dietary habits, with growing transition from home-made to eaten-out foods and pre-prepared meals (including ‘heat-and-eat’ foods, fast foods or takeaways)^(^ ^22^ ^)^. This shift has been concomitant with the current obesity epidemic, raising the possibility that these two trends are causally related. Although fast foods^(^ ^7^ ^,^ ^23^ ^,^ ^24^ ^)^ and takeaways^(^ ^22^ ^,^ ^25^ ^,^ ^26^ ^)^ have been particularly and consistently targeted as major contributors of weight gain and metabolic disorders, the associations between consumption of ready-to-eat pre-packaged dishes and diet quality and anthropometric measures have not been considered previously. A recent American study^(^ ^23^ ^)^, focused on children and young adolescents, has found that fast-food consumption is simply a by-product of a much bigger problem, related to the unhealthful Western dietary pattern and poor overall dietary habits that originate in children's homes. Furthermore, foods prepared away from home, including fast foods and store-prepared foods eaten away from home, are fuelling the increase in total energy intake^(^ ^11^ ^)^. These conclusions are largely consistent with our findings. A positive association between ready-made meal consumption and central obesity, a major indicator of body fat deposition, was recorded in the ORISCAV-LUX survey. The observed difference appeared to be largely independent of other potentially confounding lifestyle and dietary factors, including the intakes of alcoholic and non-alcoholic beverages, fruits and vegetables, added fats, grains, meats and dairy products.

BSA can provide useful information about deposits of fat in a patient's body and may provide a more accurate picture than BMI^(^ ^27^ ^)^. This measure is popular among medical practitioners for its ease of use and accurate results in clinical settings^(^ ^27^ ^)^. As it is less affected by abnormal adipose mass, BSA is a better indicator of metabolic mass^(^ ^28^ ^)^. Unlike BMI, which is intended to provide an estimate of relative weight independent of height, BSA is, by its very definition, height dependent. Therefore, it is important to note that after controlling for height, the association of high BSA with ready-made meal consumption became statistically non-significant. Clearly, height explains a significant portion of the variance in BSA. One possible explanation is that taller people are more likely to eat ready-made meals.

The accumulation of fat around the waist increases the risk of premature mortality from cancer or heart disease^(^ ^29^ ^)^. The distribution of body fat, particularly abdominal fat, is an important risk factor for obesity-related diseases. The expensive radiological imaging techniques required for precise measurement of abdominal fat content often lead the researcher to use WC as a surrogate marker of abdominal fat mass^(^ ^30^ ^)^. WC is correlated with abdominal fat mass (subcutaneous and intra-abdominal)^(^ ^31^ ^)^ and is associated with cardiometabolic disease risk^(^ ^32^ ^)^.

The consumption of ready-made meals is a specific dietary behaviour that can affect nutrient intakes and thereby diet quality. Approximately 97 % of the survey participants reported daily consumption of ready-made meals, indicating that these prepared foods have become an important part of people's diets. The consumption of ready-made meals can be linked to adverse health outcomes, particularly to metabolic obesity, through plausible mechanisms^(^ ^33^ ^,^ ^34^ ^)^. One component of pre-packaged dishes that might lead to weight gain is the use of unhealthy nutrients such as hydrogenated oils, SFA, salt, refined starchy foods and food additives to preserve flavour or enhance the taste and appearance. It is assumed that consumers of foods prepared away from home may be less knowledgeable about the energy content of foods. In addition, preparing and cooking meals at home may ensure greater control over the nutritional content and the overall quality of the food eaten.

In agreement with the results of previous studies focusing on fast foods^(^ ^23^ ^,^ ^24^ ^,^ ^35^ ^)^, in the present study, ready-made meal consumption was found to contribute to excess energy intake and poor nutritional quality of the diet, in terms of excessive amounts of fat and low levels of dietary fibre. Our findings are generally in accordance with those of other cross-sectional and prospective studies on the association between fast-food consumption and body weight^(^ ^36^ ^–^ ^38^ ^)^.

Most of the previous studies have used measures that reflect the consumption of ‘fast foods’ or ‘takeaway foods’, focusing mainly on the place of eating/purchasing^(^ ^36^ ^)^, geographical availability of takeaway foods^(^ ^22^ ^)^, and frequenting fast-food restaurants^(^ ^6^ ^)^. The present study is original in filling a gap related to ready-made meal consumption, thus enhancing the field of diet–obesity research by addressing new issues. Namely, we considered pre-cooked, ready-to-eat foods based on their contents, irrespective of whether consumed at home or away from home, as numerous other studies have suggested that the location where foods are obtained may not be as important as the nutritional quality of the food consumed^(^ ^23^ ^)^. Our definition encompassed several local and international specialties, thus providing the participants more opportunities to select accurate answers. We are not aware of any population-based study reporting an association between diet quality, obesity measures and ready-made meals, as referred to by this definition.

The present study has additional strengths. The analyses were based on a nationwide population-based dataset, collected according to standard subject selection and data collection protocols. The anthropometric parameters (outcome variables) were directly measured by trained research nurses. Detailed measurements of dietary habits were available for the analyses, leading to the possibility of extending the multivariable models to include dietary covariates and hence controlling for important additional confounders^(^ ^23^ ^)^ of the associations under investigation.

The limitations of the present study are mainly related to its cross-sectional nature, which precludes drawing conclusions about causal relationships, and the reliance on self-reported data for dietary and other lifestyle factors. As the present study focused on ‘ready-made meals’, as a predefined category in the FFQ, non-inclusion of ‘ready-to-eat’ foods, such as chicken nuggets, fish and chips, French fries, soups, entrée salads, and the many varieties of frozen dinners, would be a limitation.

From a public health standpoint, the food industry should provide healthy choices of low-energy ready-made meals, thus giving consumers more opportunities to make better choices. In parallel, efforts should be made to increase public awareness of how to read and understand food labels and to encourage restaurants to provide systematic nutritional information lists with their menus.

In conclusion, the consumption of ready-made meals is independently associated with increased abdominal obesity in adults, an indicator of central fat deposition, and the ready-made meal consumers are less likely to achieve the nutritional recommendations. In view of the high rates of both ready-made meal consumption and obesity, further follow-up data and intervention-based trials are required to fight against obesity and associated metabolic risk.

## Supplementary material

To view supplementary material for this article, please visit http://dx.doi.org/10.1017/S0007114514003468
